# Humoral anti-KLH responses in cancer patients treated with dendritic cell-based immunotherapy are dictated by different vaccination parameters

**DOI:** 10.1007/s00262-012-1263-z

**Published:** 2012-04-21

**Authors:** Erik H. J. G. Aarntzen, I. Jolanda M. de Vries, Joop H. Göertz, Marjo Beldhuis-Valkis, Huberdina M. L. M. Brouwers, Mandy W. M. M. van de Rakt, Renate G. van der Molen, Cornelis J. A. Punt, Gosse J. Adema, Paul J. Tacken, Irma Joosten, Joannes F. M. Jacobs

**Affiliations:** 1 Department of Medical Oncology, Radboud University Nijmegen Medical Centre, Nijmegen, The Netherlands; 2 Department of Tumor Immunology, Nijmegen Centre for Molecular Life Sciences, Radboud University Nijmegen Medical Centre, Nijmegen, The Netherlands; 3 Department of Laboratory Medicine, Laboratory Medical Immunology, Radboud University Nijmegen Medical Centre, Geert Grooteplein 10, 6525 GA Nijmegen, The Netherlands; 4 Department of Medical Oncology, Academic Medical Centre, Amsterdam, The Netherlands

**Keywords:** Adjuvant, Dendritic cell, Humoral response, Immunocompetence, Keyhole limpet hemocyanin, Vaccine

## Abstract

**Purpose:**

Keyhole limpet hemocyanin (KLH) attracts biomedical interest because of its remarkable immunostimulatory properties. Currently, KLH is used as vaccine adjuvant, carrier protein for haptens and as local treatment for bladder cancer. Since a quantitative human anti-KLH assay is lacking, it has not been possible to monitor the dynamics of KLH-specific antibody (Ab) responses after in vivo KLH exposure. We designed a quantitative assay to measure KLH-specific Abs in humans and retrospectively studied the relation between vaccination parameters and the vaccine-induced anti-KLH Ab responses.

**Experimental design:**

Anti-KLH Abs were purified from pooled serum of melanoma patients who have responded to KLH as a vaccine adjuvant. Standard isotype-specific calibration curves were generated to measure KLH-specific Ab responses in individual serum samples using ELISA.

**Results:**

KLH-specific IgM, IgA, IgG and all IgG-subclasses were accurately measured at concentrations as low as 20 μg/ml. The intra- and inter-assay coefficients of variation of this ELISA were below 6.7 and 9.9 %, respectively. Analyses of 128 patients demonstrated that mature DC induced higher levels of KLH-specific IgG compared to immature DC, prior infusion with anti-CD25 abolished IgG and IgM production and patients with locoregional disease developed more robust IgG responses than advanced metastatic melanoma patients.

**Conclusions:**

We present the first quantitative assay to measure KLH-specific Abs in human serum, which now enables monitoring both the dynamics and absolute concentrations of humoral immune responses in individuals exposed to KLH. This assay may provide a valuable biomarker for the immunogenicity and clinical effectiveness of KLH-containing vaccines and therapies.

**Electronic supplementary material:**

The online version of this article (doi:10.1007/s00262-012-1263-z) contains supplementary material, which is available to authorized users.

## Introduction

Keyhole limpet hemocyanin (KLH) is a high-molecular-weight glycoprotein, purified from a marine snail species called *Megathura crenulata*, that induces both cell-mediated and humoral responses in animals and humans. Because of its high immunogenicity and low toxicity, KLH is used for a variety of basic research and clinical applications [1]. KLH was introduced into the clinic in 1967 to assess immunocompetence of individuals [2]. Later, KLH became a standard carrier for the production of antibodies (Abs) to small molecule haptens such as peptides and oligosaccharides [1]. Besides this, KLH is used as a local treatment for patients with bladder cancer [3]. Finally, KLH has progressed into clinical trials as either adjuvant- or immunomonitoring tool in a variety of vaccines directed against cancer [4, 5] or infectious diseases [6].

Dendritic cells (DCs) are potent antigen presenting cells and play a central role in the induction and maintenance of antigen-specific immunity. Antigen-specific immune responses in cancer patients can be induced by exploiting autologous DCs [7]. In our institute, autologous DCs are activated ex vivo and loaded with tumor antigens. KLH is added to the vaccine both as a nonspecific T helper cell stimulus and to facilitate monitoring of the vaccine-induced immune responses [8–10]. After DC injection, the cells migrate toward the lymph nodes where they present their processed antigens to the adaptive arm of the immune system, inducing antigen-specific T and B cell responses [11].

In the last decade, several parameters of DC-based therapy have been optimized to improve vaccine-induced immunological responses, particularly in melanoma patients [12]. For example, it is well-accepted that the use of mature DC is superior to immature DC [13] and pretreatment with anti-CD25 antibodies does not improve immunological responses [14]. The route of administration largely dictates the failure or success of the induced immunological responses [15, 16]. Similarly, evidence is accumulating that the use of nonspecific helper epitopes, such as KLH, is critical to the induction of effective antitumor responses [17]. In these clinical trials, much effort has been put in the detection of tumor-antigen-specific immune responses, for example ELIspots [18, 19], T cell receptor (TCR) frequency assessment [20] and analyses of skin test biopsies [21, 22]. These assays have multiple disadvantages; the assays are laborious, have limited sensitivity, often lack association with clinical outcome and are difficult to standardize [23, 24]. Strikingly, not much attention has been paid to the dynamics, levels and patterns of the vaccine-induced anti-KLH humoral responses in clinical trials, which could provide a simple and direct method for evaluation of vaccine-induced immune responses.

Assays to monitor cellular [25, 26] and humoral [27–29] immune responses against KLH have been developed to facilitate optimal use of biomedical KLH applications. The enzyme-linked immunosorbent assay (ELISA) proved a simple and sensitive assay to measure serological Abs, and in 1979, the first quantitative assay to detect KLH-specific Ab in mice was published [27]. Five years later, the first ELISA was developed to detect KLH-specific Ab in human serum [28]. Currently, all published human anti-KLH ELISA’s report Ab responses in arbitrary units based on optical density values, which yields relative units/values only.

Because of the lack of a quantitative human anti-KLH assay, so far it has not been possible to monitor the dynamics of KLH-specific Ab responses after in vivo KLH exposure. It was neither possible to link the concentration of induced KLH Abs either to the immunocompetence of an individual or to the immunogenicity of KLH-containing vaccines.

In this study, we present the first quantitative ELISA to measure KLH-specific Abs in human serum, measuring both IgG, IgA, IgM and all IgG-subclasses. We demonstrate that it is now possible to monitor the dynamics of both KLH-specific Ab levels and Ab class-switching in individuals who are repeatedly exposed to KLH. Furthermore, we retrospectively analyzed the humoral anti-KLH responses of melanoma patients enrolled in our clinical trials investigating dendritic cell-based vaccinations. Our data confirm that individuals exposed to KLH react to it as a primary immunogen, inducing a characteristic primary immune response. Moreover, analyses of our clinical DC-based vaccination protocols demonstrated that variations in the vaccination parameters dramatically influence both the quantity and the quality of the induced humoral response.

## Materials and methods

### Preparation of serum pool containing anti-KLH antibodies

Serum was pooled from HLA-A*02:01 melanoma patients and colorectal cancer patients who were previously vaccinated with KLH-pulsed dendritic cells (DCs). To generate a serum pool with a broad spectrum of KLH-specific Abs, sera were obtained from 28 patients enrolled in different clinical trials within our institute in which the maturation status of the DCs and the route of vaccine administration varied per clinical trial [13–15, 30, 31]. For the exact details regarding the vaccination protocols and the patient inclusion, we refer to these individual studies. In all studies, KLH was added at day 3 of the DC culture at a concentration of 10 μg/ml and patients received maximum three vaccination cycles of 3 bi-weekly vaccinations at a 6-month interval. Informed consent was obtained from all patients, and the study was approved by our Institutional Review Board.

### Purification of KLH-specific antibodies

Using a KLH-coated column (Alpha Diagnostic International, San Antonio, TX, USA), 18 ml starting serum pool of KLH-specific Abs was isolated from the above-mentioned trials. Prior to use, the column was extensively washed with elution buffer (0.1 M glycine HCl pH 2.5) to dissociate multimeric KLH and remove monomers that were not covalently linked to the Sepharose bed. Subsequently, the column was equilibrated with PBS, followed by application of the pooled serum, washed with PBS and elution of the KLH-specific Abs with elution buffer and neutralized with Tris/HCL pH 9.0. The KLH-specific IgM-, IgA- and IgG-Abs in the eluate with a volume of 1,380 μl were quantified using nephelometry on an Immage apparatus (Beckman Coulter, Brea, CA, USA), IgG subclasses were measured on a BNII (Siemens, Münich, Germany) using reagents of The Binding Site (Birmingham, UK) all according to the manufacturers protocol. Following this approach, a 1.4 ml polyclonal anti-KLH stock was obtained in which all major immunoglobulin isotypes and IgG subclasses were represented (Supplementary Table 1, available online). This stock of purified and quantified KLH-specific Abs was stored at −80 °C and used to generate standard curves for the quantitative ELISA. All experiments in this manuscript were performed with one single calibrated work standard of 34 ml created from 28 different patients.

### Quantitative anti-KLH ELISA

Ninety-six-well plates were coated for 18 h at 4 °C with 5 μg/mL KLH (Biosyn, Carlsbad, CA, USA) in PBS in a total volume of 120 μL per well, and wells were subsequently blocked with skimmed milk powder. After washing the plates, different concentrations of patient serum were added for 1 h at room temperature. To prepare suitable standard curves for the anti-KLH ELISA’s, the work standard was serially diluted to establish a 7-point standard curve for each Ab isotype (Supplementary Figure 1, available online). For analysis of individual patient samples, a standard curve was added to each plate. After washing, specific Abs against human IgM or IgA or total IgG or IgG1 or IgG2 or IgG3 or IgG4 labeled with horseradish peroxidase (Invitrogen, San Diego, CA, USA) at 1:500 were added to the wells as 100 μL aliquots. The isotype-specific secondary Abs allow quantification of both human IgM, IgA, total IgG, IgG1, IgG2, IgG3 and IgG4 KLH-specific Abs. After a further incubation for 1 h at room temperature and a final washing with buffer, peroxidase activity was revealed using 3,3′ 5,5-tetramethyl-benzide as substrate. The reaction was stopped with sulfuric acid, and samples were measured in a microtiter plate reader at 450 nm. A uniform detection limit of the anti-KLH ELISA’s was arbitrarily determined at 20 μg/ml. This detection limit falls within the lower linear part of all anti-KLH calibration curves. Precision and linearity were determined according to the Clinical and Laboratory Standards Institute protocols EP5 and EP6, respectively.

### Statistical analysis

Precision and linearity were analyzed using EP Evaluator 9 software, and all other statistical analyses were performed by Graphpad Prism 5.0. For the comparison of different vaccination protocols, patients were grouped based on similar protocol characteristics, such as stage of disease on entry, route of administration, pretreatment with anti-CD25 antibodies, additional treatment with interleukin-2 (IL-2) or the method of tumor antigen loading. For the comparison of 2 groups, all the above-mentioned protocol characteristics should be similar, except for the single parameter under investigation. Maximum KLH-specific Ab responses, out of at least 3 samples obtained at standard time points during the first cycle, were compared, as this reflects the individual’s competence to generate a humoral response. Nonparametric Mann–Whitney test was performed to compare the KLH-specific Ab responses of similar isotypes in two groups of melanoma patients exposed to KLH. A two-tailed *p* value <0.05 was considered significant.

## Results

### Assay performance and validation

**Fig. 1:**
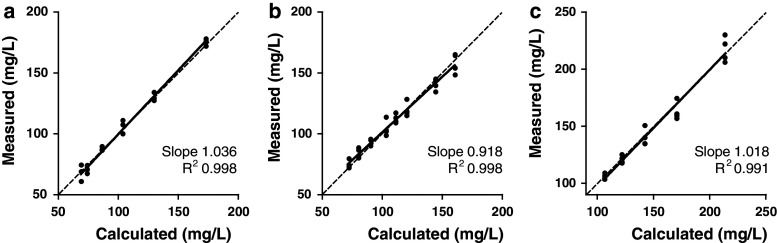
Serial dilution linearity of the anti-KLH ELISA*.* Serum samples of patients exposed to KLH were serially diluted with assay buffer and measured in quadruplicate. The results for linearity of the ELISA for the anti-KLH isotypes IgG (**a**) IgA (**b**) and IgM (**c**) are shown. Calculated concentrations are based on stock-concentration and dilution factor. Slopes and coefficients of determination (R^2^) are indicated in each panel. The *line* of identity is indicated by the *dashed line*

To evaluate the precision of the assay, pooled serum samples were measured in 2 replicates per run, 1 run per day for a minimum of 20 runs. The resulting anti-KLH ELISA’s have an intra-assay imprecision, denoted by the coefficient of variation (CV), that ranged from 4.3 to 6.7 %. The inter-assay CV varied from 6.4 to 9.9 % (Table 1). To test for assay-linearity, we serially diluted 2 individual patient serum samples per KLH assay isotype at a minimum of 5 levels, assayed in quadruplicate. The results yielded slopes ranging from 0.918 to 1.036, and the coefficients of determination (R^2^) ranged from 0.991 to 0.998 (Fig. 1). A method-comparison analysis was not possible, as there is no golden standard quantitative human anti-KLH available.

**Table 1:** Performance assessment of the anti-KLH ELISA assays

	IgG_total_		IgG_1_		IgG_2_		IgG_3_		IgG_4_		IgA_total_		IgM_total_	
Mean (mg/L)	478.0		282.0		80.4		5.0		76.3		22.8		719.7	
Variation	Intra	Inter	Intra	Inter	Intra	Inter	Intra	Inter	Intra	Inter	Intra	Inter	Intra	Inter
SD	22.1	30.5	13.6	25.1	5.4	7.1	0.22	0.40	4.1	5.1	1.1	2.2	44.4	71.6
CV (%)	4.6	6.4	4.8	8.9	6.7	7.1	4.3	7.9	5.4	6.7	5.0	9.7	6.2	9.9

CV, coefficient of variation; intra, intra-assay imprecision; inter, inter-assay imprecision

### Monitoring the dynamics of humoral anti-KLH responses in individual patients

**Fig. 2:**
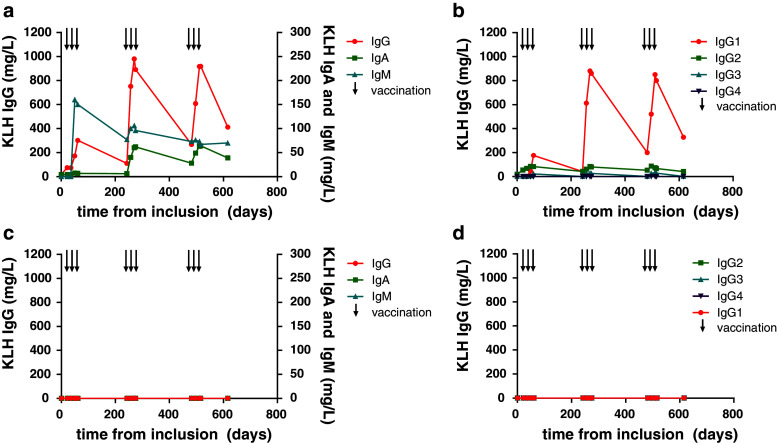
A detailed characterization of KLH-specific antibody responses in individual patients, two examples. Two patients who received 9 vaccinations of KLH-loaded dendritic cells over a period of 18 months are characterized in detail for KLH-specific antibody responses. Each vaccination is indicated by a *black arrow*. One patient had a robust humoral response, the kinetics of the KLH-specific IgG, IgA and IgM responses are shown in (**a**). In **b,** the IgG immune response is subdivided into the four IgG subclasses, demonstrating that IgG1 predominantly contributes to the KLH-specific immune response in this patient. The second patient received vaccinations with DC not loaded with KLH. In this patient, KLH-specific antibody responses were completely absent (**c**, **d**)

Repetitive serum sampling of individual patients enrolled in clinical trials on dendritic cell-based vaccinations allows in-depth monitoring of the kinetics of the KLH-specific Ab responses during therapy. In the majority of patients, we detected the first KLH-specific Abs after the 2nd or 3rd vaccination. We show one representative melanoma patient who had detectable levels of KLH-specific IgM Abs after the second vaccination (Fig. 2a). The IgM response was followed by KLH-specific IgG Abs and after four vaccinations also by IgA Abs. Ab titers were drastically decreased between vaccination cycles and after the last vaccination.

**Fig. 3:**
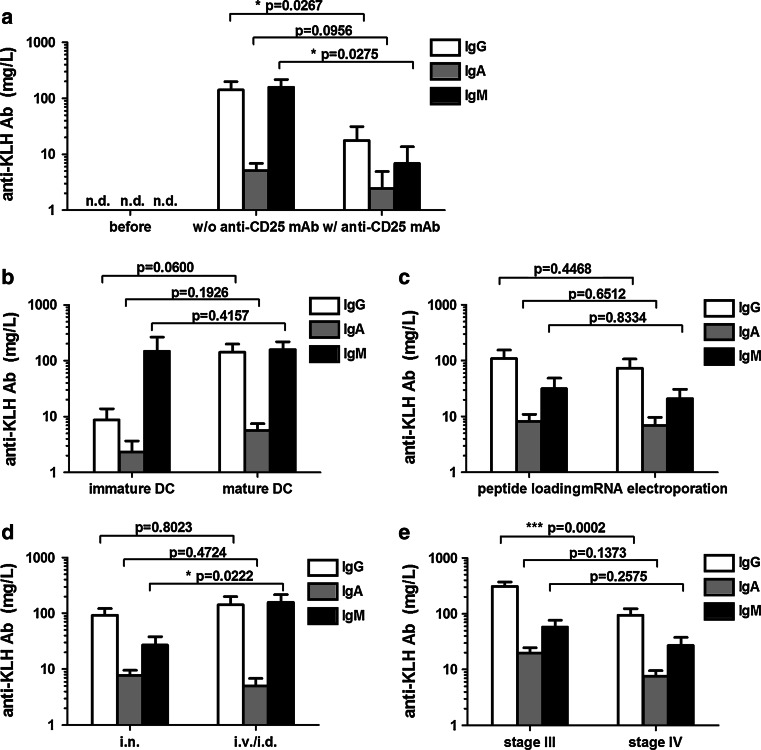
Variations in vaccination parameters induce different humoral anti-KLH responses*.* In total, 128 melanoma patients were exposed to KLH by 3 bi-weekly vaccinations containing KLH-loaded DC. None of the 35 patients tested (protocols 4 and 5) had KLH-specific antibodies prior to vaccination (**a**). KLH-specific Ab responses compared between **a** patients treated with or without daclizumab prior to the KLH exposure; **b** patients vaccinated with immature or mature KLH-loaded DC; **c** peptide-loaded or mRNA-transfected KLH-loaded DC; **d** intranodal or intravenous/intradermal routes of administration and **e** patients with locoregional metastatic disease or distant metastatic disease at inclusion. Mean levels of KLH-specific IgG (*white bars*), IgA (*gray bars*) and IgM (*black bars*) antibodies are shown in mg/L. *Error bars* indicate standard error of the mean; *Nd* not detected

IgG subclass analysis showed that IgG1 predominantly contributed to the KLH-specific IgG response in this patient (Fig. 2b). This example demonstrates that the KLH Ab response strongly depends on the schedule of KLH exposure and the time point of serum sampling. One vaccinated patient received 9 vaccinations that did not contain KLH, because of a severe shellfish allergy. We detected no KLH-specific Abs in this specific patient (Fig. 2c, d), demonstrating that KLH-specific Ab responses are caused specifically by the KLH added to the vaccine and not by the vaccine itself. The high specificity of the anti-KLH ELISA is further illustrated by the fact that none of the 57 tested patients had detectable KLH-specific Abs prior to vaccination (Fig. 3a, bars indicated by ‘before’).

### Variations in vaccination parameters have major influence on the levels and patterns of humoral anti-KLH responses

Another application of this novel assay is that it allows the comparison of the humoral immunogenicity of different KLH-containing interventions. To demonstrate this, we performed subgroup analyses in a total of 118 melanoma patients, 43 stage III and 85 stage IV, who received dendritic cell-based vaccinations according to different protocols (Table 2).

**Table 2:** Patients and protocol characteristics

Protocol nr.	Stage	Nr of patients	Maturation status	Route of administration	Anti-CD25 pretreatment	Method of Ag-loading
1	IV	9	Immature	i.v./i.d.	No	Peptide pulsing
2	IV	17	Mature	i.n.	No	mRNA electroporation
3	IV	24	Mature	i.n.	No	Peptide pulsing
4	IV	13	Mature	i.v./i.d.	Yes	Peptide pulsing
5	IV	22	Mature	i.v./i.d.	No	Peptide pulsing
	Total	85				
6	III	43	Mature	i.n.	No	Peptide pulsing or mRNA electroporation
	Total	43				

Pretreatment with a single dose of intravenous humanized monoclonal anti-CD25 antibody (Daclizumab) significantly reduced the overall anti-KLH Ig responses (Fig. 3a). The mean level of anti-KLH IgG without pretreatment (protocol 5, *n* = 22) was 142 mg/L compared to 18 mg/L after pretreatment (protocol 4, *n* = 13, *p* = 0.0267). With regard to anti-KLH IgA, the mean levels were 5 mg/L with no pretreatment and 2 mg/L with pretreatment; for anti-KLH IgM, this was 157 mg/L compared to 7 mg/L, respectively (*p* = 0.0275).

Vaccination with immature DC (protocol 1, *n* = 9) barely induced anti-KLH antibodies compared to vaccination with mature DC (protocol 5, *n* = 22) (Fig. 3b). Anti-KLH IgG antibodies were detected in 2/9 patients upon immature DC vaccination and in 12/22 patients after vaccination with mature DC, mean 9 mg/L and 142 mg/L, respectively (*p* = 0.0600). Anti-KLH IgM antibodies were induced in the same patients, 2/9 patients after immature DC vaccination and 9/22 patients after mature DC vaccination, to similar extend, 147 mean mg/L and 157 mg/L, respectively (*p* = 0.4157).

As expected, the method of loading the tumor antigens onto the dendritic cells, either pulsing with defined peptides (protocol 3, *n* = 24) or electroporation with mRNA encoding the tumor antigens (protocol 2, *n* = 17), did not affect the concentration or the pattern of vaccine-induced anti-KLH humoral responses (Fig. 3c).

Combined intravenous/intradermal vaccination (protocol 5, *n* = 22) induced significantly more anti-KLH IgM antibodies as compared to intranodal vaccination (protocol 2 and 3, *n* = 41), mean 157 mg/L and 27 mg/L, respectively (*p* = 0.0222), but no differences in IgG or IgA levels were noted (Fig. 3d).

We observed significantly higher anti-KLH IgG titers in melanoma patients who were vaccinated adjuvant to radical lymph node dissection (stage III) compared to melanoma patients with irresectable locoregional disease or distant metastatic disease (stage IV). Mean concentrations of all isotypes were at least two times higher in stage III patients compared to stage IV melanoma patients (Fig. 3e). However, the most prominent was anti-KLH IgG, mean 310 mg/L in stage III patients (protocol 6, *n* = 43) compared to 94 mg/L in stage IV patients (protocol 2 and 3, *n* = 41), *p* = 0.0002.

## Discussion

KLH is widely used for biomedical applications because of its remarkable immunogenic properties. In the current study, we describe a novel, highly sensitive and quantitative, sandwich ELISA standardized to measure IgM, IgA and IgG Abs specific for KLH. We demonstrate that it is now possible to monitor the dynamics and concentrations of humoral KLH-specific immune responses in individuals exposed to KLH and in clinical trials. Importantly, we show that vaccine-induced KLH-specific humoral responses largely depend on variations in vaccination parameters in DC-based clinical trials.

Using our novel anti-KLH ELISA, we found that melanoma patients exposed to KLH-loaded DCs often initially develop KLH-specific IgM Abs, which is associated with a primary immune response as was expected from the literature [32]. After subsequent vaccinations in our patients, this is usually followed by class-switching and the production of KLH-specific IgG Abs, suggestive for a secondary immune response. This secondary immune response is accompanied by KLH-specific IgA Abs in a minority of patients. In general, Ab-isotype switching and the identity of the immunoglobulin subclasses is regulated by cytokines and B cell activators [33]. In this respect, Ab responses in terms of isotype and subclass will be influenced by the route and conditions under which KLH is administered. In our studies, in which all individuals were exposed to KLH-loaded cytokine-matured DCs, IgG1 was the predominant subclass produced. We hypothesize that the method of KLH exposure and/or individual patient characteristics can result in significant skewing of the IgG subclass response.

We further demonstrate that, using this novel assay, it is now possible to link the concentration of induced KLH-specific Abs to the humoral immunogenicity of the KLH-containing DC-based vaccines used in our institute. In our cohort of patients with metastatic melanoma, we have found that adjustments in the vaccination protocol can significantly affect the humoral responses against KLH. We show for example that a single dose of daclizumab, a humanized monoclonal anti-CD25 Ab, prior to the injection of KLH-loaded DCs almost completely abolishes the KLH-specific humoral response. Blocking CD25 on activated T cells and regulatory T cells greatly reduces the capacity of the immune system to respond to de novo antigens. The in vivo effects of daclizumab on T cells are well documented [34], but the effects of daclizumab on humoral immune responses were largely unexplored. Although injected prior to vaccination, circulating daclizumab may bind to CD25-expressing vaccinated DC, providing yet another possible explanation for the strongly reduced vaccine-induced humoral responses to KLH. The maturation status of the DCs is another parameter that strongly affects humoral immunogenicity of the vaccine. We previously demonstrated that more effective cellular immune responses are induced with mature DCs compared to immature DCs [13]. Now we demonstrate that mature DC also generate stronger humoral immune responses and are essential for efficient immunoglobulin class-switching. In addition, we show that there is no significant difference in the KLH-specific IgG and IgA responses when the KLH-vaccine is administered intravenously/intradermally or intranodally in melanoma patients. However, intravenous/intradermal injections tend to result in increased KLH-specific IgM responses compared to intranodal injection. In case of differential tumor antigen loading onto the DC, we would not expect different KLH responses since in all protocols, the DC are pulsed with KLH at day 3 after isolation, and loading with tumor antigen occurs just before administration. Indeed, with regard to different methods of tumor antigen loading, we did not detect differences in levels or specificity of anti-KLH humoral responses.

Several lines of evidence demonstrate that tumor cells actively create a local and systemic immune suppressive environment [35–37]. The level of tumorload may therefore negatively correlate with the competence of a patients’ immune system to respond to antigens such as KLH [38]. We investigated the humoral KLH responses in patients treated with DC-based vaccinations adjuvant to radical lymph node dissection, and thus without macroscopic disease (stage III), and in patients with distant metastatic and measurable disease, stage IV melanoma. We indeed confirm that anti-KLH Ab production was higher in stage III patients compared to patients with stage IV melanoma, which argues in favor of applying immunotherapy at earlier stages of disease.

Further analysis is warranted to investigate whether the KLH-specific Ab response in the context of anti-cancer vaccines can be used as a surrogate biomarker for tumor-specific immune responses. The standardized ELISA presented here can facilitate in this effort as it allows inter-institutional comparisons of the humoral immunogenicity of KLH-containing vaccines.

Although we initially developed the quantitative anti-KLH ELISA to monitor vaccine-induced immune responses, the assay allows quantification of humoral responsiveness as a measure of immunocompetence in individuals exposed to KLH. Since KLH elicits a primary immune response, intramuscular injection of KLH is the method of choice to investigate the capacity of an individual to respond to novel antigens, overcoming the confounding influence of previous exposure history [39].

Finally, because of its capacity as a nonspecific immune stimulator, KLH has been investigated as an intravesical agent to treat superficial bladder cancer [3]. Previously, it has been reported that the presence of anti-KLH antibodies was associated with treatment response [40]. In this respect, it is interesting to investigate whether in-depth analysis of the KLH-specific Ab responses in these patients can be an early predictor of therapy outcome.

In conclusion, we here present a sensitive and specific quantitative assay to measure KLH-specific Abs in human serum. We demonstrate that with this assay, it is now possible to monitor both the dynamics and absolute concentrations of KLH-specific humoral responses in individuals exposed to KLH, for example to evaluate humoral immune competence. In addition, it may provide a valuable biomarker for the immunogenicity and clinical effectiveness of KLH-containing vaccines and therapies.

### Electronic supplementary material

Below is the link to the electronic supplementary material.

Supplementary material 1 (PDF 45 kb)
