# Supplementary material for: Humoral anti-KLH responses in cancer patients treated with dendritic cell-based immunotherapy are dictated by different vaccination parameters
Source: Cancer Immunol Immunother. 2012 Apr 21;61(11):2003–11. doi: 10.1007/s00262-012-1263-z (PMC3493659; doi:10.1007/s00262-012-1263-z)
Supplement: Supplementary file 1 — Supplementary material 1 (PDF 45 kb) [file 262_2012_1263_MOESM1_ESM.pdf]

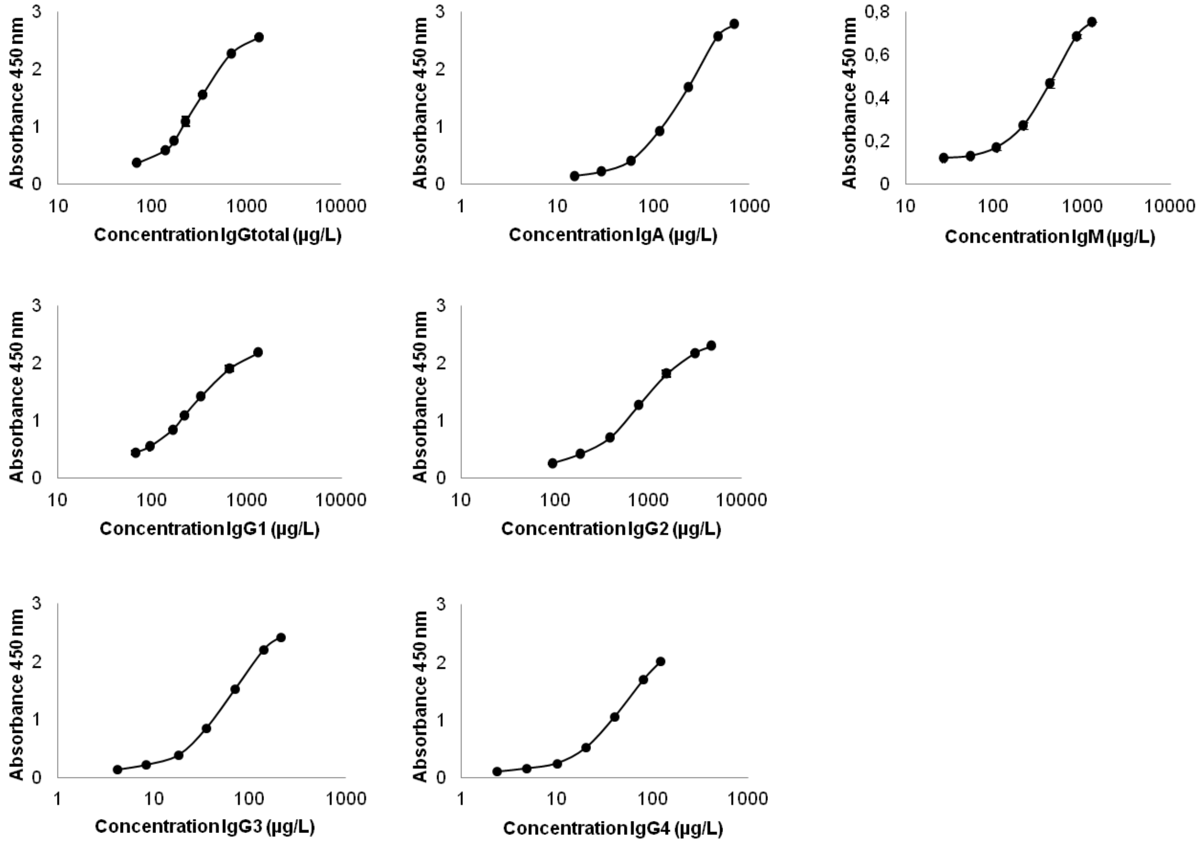

Supplementary Fig. 1 *Anti-KLH antibody isotype-specific standard curves.*

A pool of purified KLH-specific human antibodies is serially diluted to establish isotype-specific 7-point standard curves. Dilutions were as followed, for IgG total, IgG1 and IgG4: 300x, 600x, 1200x, 1800x, 2400x, 4200x and 6000x; for IgG2, IgG3 and IgA: 50x, 100x, 150x, 250x, 500x, 750x and 1000x; for IgM: 60x, 120x, 240x, 360x, 480x, 600x and 1200x. The horizontal axis indicates the absolute concentration in µg/L not adjusted for the dilution step preceding the measurement.

Supplementary Table 1 KLH-specific Abs after column-purification of pooled serum of patients exposed to KLH

**Abs in eluate**	**Concentration**
IgG_{total}	1.370 mg/L
IgG_1	990 mg/L
IgG_2	470 mg/L
IgG_3	21 mg/L
IgG_4	12 mg/L
IgA	70 mg/L
IgM	130 mg/L

Abbreviations: Abs, antibodies
